# Strategies for Optimizing Growth in Children With Chronic Kidney Disease

**DOI:** 10.3389/fped.2020.00399

**Published:** 2020-07-30

**Authors:** Dieter Haffner

**Affiliations:** Department of Pediatric Kidney, Liver and Metabolic Diseases, Hannover Medical School Children's Hospital, Hanover, Germany

**Keywords:** chronic kidney disease, children, growth, growth hormone, nutrition, CKD-MBD

## Abstract

Growth failure is a hallmark in children with chronic kidney disease (CKD). Therefore, early diagnosis and adequate management of growth failure is of utmost importance in these patients. The risk of severe growth retardation is the higher the younger the child is, which places an additional burden on patients and their families and hampers the psychosocial integration of these children. Careful monitoring of growth, and effective interventions are mandatory to prevent and treat growth failure in children with CKD at all ages and all stages of kidney failure. Early intervention is critical, as all therapeutic interventions are much more effective if they are started prior to the initiation of dialysis. Prevention and treatment of growth failure focuses on: (i) preservation of renal function, e.g., normalization of blood pressure and proteinuria by use of inhibitors of the renin-angiotensin aldosterone system, (ii) adequate energy intake, including tube feeding or gastrostomy in case of persisting malnutrition, (iii) substitution of water and electrolytes, especially in children with renal malformation, (iv) correction of metabolic acidosis, (v) control of parathyroid hormone levels within the CKD-dependent target range, (vi) use of recombinant human growth hormone in cases of persistent growth failure, and, (vii) early/preemptive kidney transplantation using steroid-minimizing immunosuppressive protocols in children with end-stage CKD. This review discusses these measures based on recent guidelines.

## Introduction

Growth failure is a hallmark of children with chronic kidney disease (CKD). Although height prognosis has improved considerably in recent decades, ~40% of children requiring kidney replacement therapy before puberty continue to achieve a reduced adult height (< −2 SD) (1–8). The risk of severe growth retardation is the higher the younger the child is, which places an additional burden on patients and hampers their psychosocial integration (9). In addition, short stature is associated with markedly enhanced mortality, supporting the concept that normal longitudinal growth is a sensitive indicator of overall well-being in these patients (10–12).

**Figure 1:**
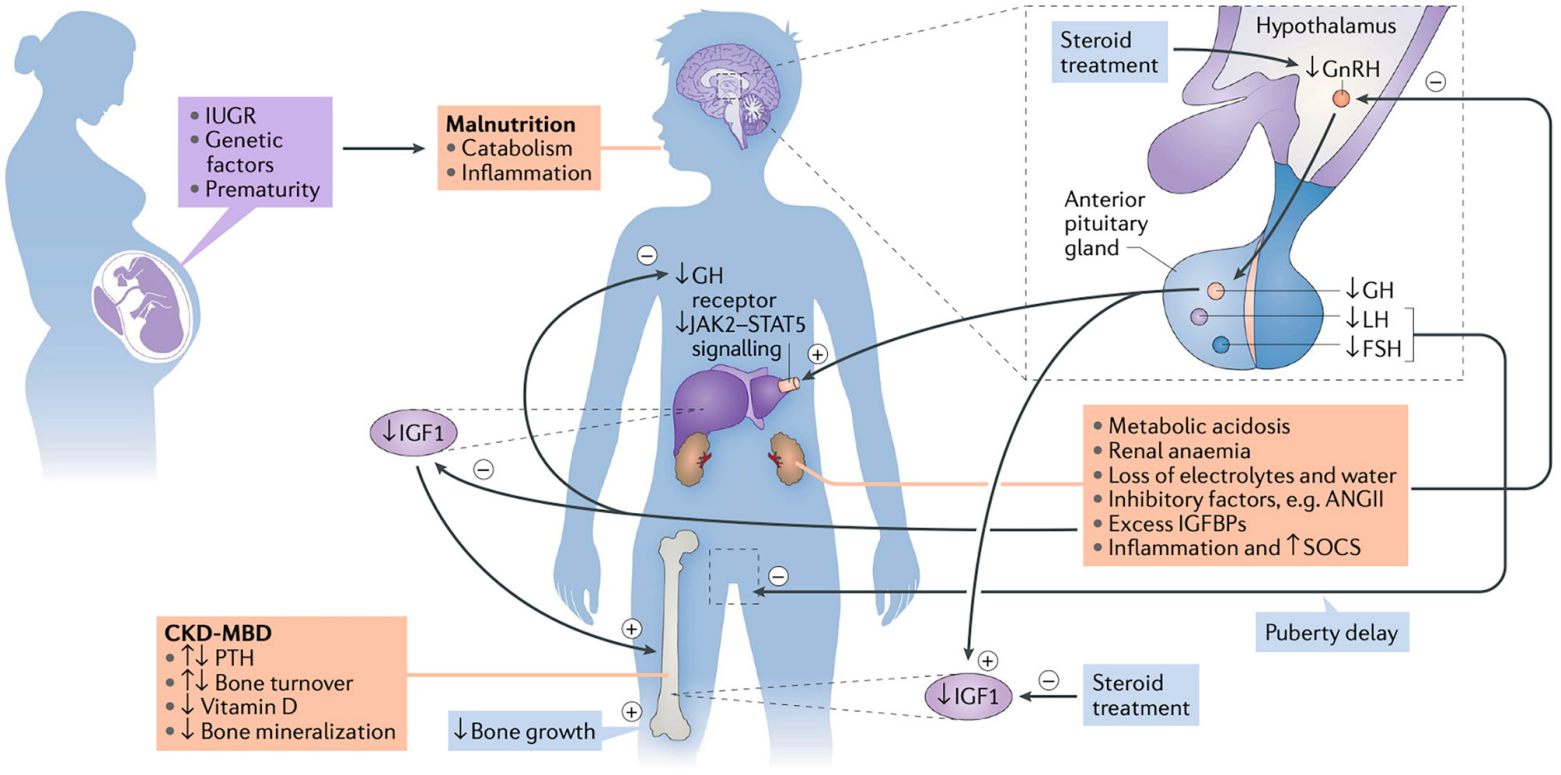
The etiology of growth failure in CKD is multifactorial and includes intrauterine growth restriction (IUGR), genetic factors, such as parental height and primary kidney disease, prematurity and malnutrition which especially limits growth in children with congenital CKD. Mineral and bone disorder (CKD-MBD), metabolic acidosis, anemia, loss of electrolytes and water, and disturbances of the somatotropic and gonadotropic hormone axes are additional factors. CKD is a state of growth hormone (GH) insensitivity, characterized by a deficiency of functional insulin-like growth factor I (IGF-I), reducing GH receptor expression in target organs like the liver, disturbed GH receptor signaling via the Janus kinase signal transducers, activators of transcription (JAK2-STAT5) due to inflammation-induced SOCS (suppressor of cytokine signaling), and increased IGF-binding capacity due to excess of IGFBPs. Finally, reduced release of hypothalamic gonadotropin-releasing hormone (GnRH), due to uremia-related inhibitory factors such as angiotensin II (AngII) and steroid treatment, may result in decreased circulating levels of bioactive luteinizing hormone (LH), hypogonadism and reduced pubertal growth spurt (13). PTH, parathyroid hormone; FSH, follicle stimulating hormone; IGFBP, insulin-like growth factor binding proteins. Figure and figure legend taken from (13).

The pathogenesis of short stature in CKD is multifactorial. Chronic kidney disease may be caused by acquired or congenital kidney abnormalities, that manifest themselves in early or late childhood and vary greatly in severity and rate of progression. Similarly, a broad spectrum of concomitant complications (e.g., poor nutritional intake, metabolic acidosis, and electrolyte disturbances), as well as CKD- associated growth hormone (GH) insensitivity, has to be considered (Figure 1). While some factors, such as nutritional and hormonal abnormalities, hematological and metabolic disorders, such as electrolyte imbalance, acidosis, mineral and bone disorder (CKD-MBD) and anemia are potentially correctable, the effects of others, birth parameters, associated syndromes and parental height, are not (14). A major breakthrough in the management of uremic growth failure is the introduction of recombinant human growth hormone (rhGH), which overcomes CKD-associated GH insensitivity and thereby increases growth outcome in children with CKD (15). A detailed review on growth and nutrition in pediatric CKD was recently published in this journal (16). This article discusses the main measures to optimize growth in children with CKD during the various stages of kidney disease, i.e., prior to dialysis, on dialysis, and after kidney transplantation, based on published guidelines.

## Monitoring

Adequate monitoring of growth in children with CKD matters. Height in these children may rapidly descend across the percentiles, especially during periods of expected high growth rates, i.e., infancy, early childhood and adolescence. In fact, height losses of up to 1.0 SD per year were reported in infants on dialysis (17). “As with any child who has a potentially growth-limiting chronic disease, length (up to the age of 2 years) and height (from 2 years) should be regularly evaluated by trained personnel, using calibrated equipment and standardized techniques” (13). Anthropometric data should be compared with gender- and age-specific reference values for healthy children. The gestational age should be taken into account in assessing the length of prematurely born infants. Young children and those with more advanced CKD should be seen more often than older children and those with only mild kidney function impairment (Table 1). “Children with evidence of growth retardation, comorbidities, including central nervous system or involvement of the liver or heart, require more frequent assessment than those with a milder or more stable disease” (13). “The calculation of annual height velocity allows for identification of children with reduced growth rates, i.e., below the 25th percentile, and thus identifies children, who may benefit from growth-promoting measures (vide infra)” (13). “By contrast, a height velocity above the 75th percentile indicates catch-up growth” (13). Current national growth charts should be referred to. Alternatively, regional as well as international growth charts may be used. As with any child, growth potential is related to parental height and the genetic target height should be calculated using Tanner's formula for girls and boys:

$$\begin{array}{l} Girls:\;\left(height_{\text{mother}}+height_{\text{father}}\;-13\right)/2\\ Boys:\;\left(height_{\text{mother}}+height_{\text{father}}\;+13\right)/2 \end{array}$$

These formulas should not be used in cases of parents with chronic disease, whose height may be impaired. Unfortunately, the application of height predicting models based on bone age cannot be recommended in children with CKD, as they, largely, overestimate adult height (18).

**Table 1:** Recommended assessment intervals (in months) of length/height and length/height velocity by CKD stage and age.

	**CKD stage**			
	**3**	**4**	**4–5**	**5D**
**Length/height**				
Age 0–1 years	0.5–2	0.5–2	0.5–2	0.5–2
Age 1–3 years	1–3	1–2	1–2	1–2
Age >3 years	3–6	1–3	1–3	1–3
**Length/height velocity**				
Age 0–1 years	0.5–2	0.5–2	0.5–2	0.5–1
Age 1–3 years	1–6	1–3	1–3	1–2
Age >3 years	6	6	6	6

*CKD, chronic kidney disease; table taken from (13)*.

## Prevention and Treatment

### Preservation of Renal Function and Dialysis

Growth retardation is rarely seen in CKD stages 1–2, often noted in CKD stages above 3, and is generally more pronounced in children on dialysis, compared to patients prior to dialyis (19). This may, at least partly, be due to the negative association between GH-insensitivity and glomerular filtration rate (GFR) (20). The logical consequence of these findings is to keep up GFR rates and provide adequate dialysis in children requiring maintenance dialysis (Table 2). Preservation of renal function requires treatment of elevated blood pressure, with the goal of blood pressure values below the 50th and 75th percentile in proteinuric and non-proteinuric children, respectively (24). Renin-angiotensin aldosterone system inhibitors, preferentially angiotensin-converting enzyme inhibitors or angiotensin receptor inhibitors, should be used to treat high blood pressure and ameliorate proteinuria in children with CKD (25, 26). Nephrotoxic medication should be avoided, and urinary tract infections in children with congenital abnormalities of the kidneys and urinary tract (CAKUT) should be treated.

**Table 2:** Main measures for prevention and treatment of growth failure in pediatric CKD.

Prevention: • Close growth monitoring with intervals depending on previous growth, age and stage of CKD. • Preserve renal function by: ° Treating elevated blood pressure and reducing proteinuria, preferrably using RAAS inhibitors. ° Avoiding nephrotoxic medication. ° Prompt treatment of urinary tract infections. • Provide adequate energy and protein intake and consultation with a renal dietician. ° “Consider enteral feeding by gastrostomy or nasogastric tube in cases of persistent insufficient oral intake” (21). • Substitute water and electrolyte losses and correct metabolic acidosis. • “Keep PTH levels in the recommended CKD stage-dependent target range and substitute native vitamin D in cases of low vitamin D levels” (22, 23). • Aim for early (preemptive) renal transplantation with minimal steroid exposure in patients with end-stage CKD. • Provide adequate dialysis in patients requiring maintenance dialysis.
Treatment: • “Consider use of growth hormone treatment in cases of persistent growth failure, i.e., height <3rd percentile and height velocity <25th percentile, excluding patients who have received a transplant within the last 12 months” (13). • “Consider intensified dialysis or hemodiafiltration in in patients requiring maintenance dialysis presenting with persistent growth failure” (13). • “Consider use of rhGH therapy in pediatric kidney transplant recipients for whom expected catch-up growth cannot be achieved by steroid minimization, or for patients where steroid withdrawal is not feasible due to high immunological risks, particularly in children with suboptimal graft function (GFR < 50 ml/min/1.73 m^2^)” (13).

*CKD, chronic kidney disease; RAAS, renin-angiotensin aldosterone system; PTH, parathyroid hormone*.

There is increasing evidence that intensified dialysis, achieved either through extended thrice weekly, nocturnal or short daily sessions, are effective measures to improve growth in short children presenting with persistent growth failure while on conventional dialysis (27, 28). In addition, a recent non-randomized study demonstrated superior growth in children treated with hemodiafiltration compared to those with conventional hemodialysis (29). “Therefore, initiation of intensified dialysis or hemodiafiltration should be considered in children on maintenance dialysis presenting with persistent growth failure” (13).

### Nutrition

The assurance of adequate caloric intake is of major importance to prevent CKD-associated growth failure, especially in infants and young children (21). This requires the patient and families to be advised by a renal dietician, especially when supplementary feeding via a nasogastric or gastrostomy tube is required (21). Physicians should be proactive in instituting tube feeding in any infant or young child not achieving adequate energy intake, as this may result in suboptimal growth outcome (21). “In general, the initial prescription for energy intake in children with CKD should approximate that of healthy children of the same age (suggested dietary intake, SDI)” (21). “To optimize growth in children with suboptimal weight gain and linear growth, energy intake should be adjusted toward the higher end of the SDI” (21). “In addition; to promote optimal growth, target protein intake in children with CKD should be at the upper end of the SDI” (21).

### Correction of Acid-Base/Electrolyte Abnormalities

Metabolic acidosis is associated with poor growth in children with CKD. Therefore, metabolic acidosis should be corrected aiming for serum bicarbonate levels equal to or above 22 mEq/L. This can be assured by treatment with sodium bicarbonate and/or the use of HCO_3_-based or lactate-based dialysis solutions in patients on dialysis (30). Supplementation of water and/or electrolytes is often required in poliuric patients and those with salt-losing nephropathies. It is important to note, that young children on peritoneal dialysis often require supplementation with large amounts of sodium chloride, since considerable losses (i.e., 2–5 mmol/kg body weight) via peritoneal ultrafiltration may occur.

### Treatment of Renal Osteodystrophy

Although severe secondary hyperthyroidism may result in growth arrest, only a weak association between parathyroid hormone (PTH) values and linear growth was reported in children with CKD and recommended CKD-stage dependent PTH target ranges differ widely (31, 32). “In general, minimal PTH suppressive calcitriol dosages should be used in order to keep PTH levels within the desired target range” (22). “Supplementation with cholecalciferol or ergocalciferol should be initiated in children with serum 25-hydroxyvitamin D concentrations below 75 nmol/L (<30 ng/mL)” (23).

### Physical Activity

Physical activity is often reduced in children with CKD and is associated with abnormalities in serum markers of bone formation and remodeling (33). Although endurance exercise improves bone formation in subtotal nephrectomized young rats (34), studies demonstrating better growth after increased physical activity in children with CKD are lacking.

### Treatment of Renal Anemia

Although longstanding anemia in pediatric CKD patients may result in anorexia and catabolism, its impact on growth is small but should nevertheless be treated to improve overall well-being, physical capacity and cardiovascular health (19, 35, 36).

### Growth Hormone Treatment

Treatment with recombinant human growth hormone (rhGH) is a measure proven to improve adult height in short children with CKD (13). The positive results from initial clinical trials have been confirmed by several large, prospective, observational studies and registries, underlining the importance of its use in this population in order to improve growth outcome (19, 37–41). Clinical practice recommendations for the use of rhGH in pediatric CKD patients have been provided by expert committees (13, 42). Infancy and early childhood are the most sensitive phases for the growth-suppressing effects of CKD which is at least partly related to GH insensitivity. Any impairment of height velocity during these phases may result in profound growth retardation. In addition, catch-up growth after correction of reversible causes of growth failure and initiation of other measures, including rhGH, may not be complete with the potential consequence of irreversible loss of growth potential (13). RhGH induced catch-up growth is positively associated with estimated glomerular filtration, mid-parental height, initial target height deficit and duration of rhGH therapy, and negatively correlated with patient age (8, 43–45). “Therefore, rhGH should be initiated as soon as growth retardation becomes evident” (13).

The presence of open epiphysis on the X-ray of the left wrist indicates growth potential, which is a prerequisite for the indication of rhGH treatment in children presenting with persistent short stature (13). “Children above 6 months of age with stages 3–5 CKD or on dialysis should be candidates for rhGH therapy if they have persistent growth failure—defined as a height below the 3rd percentile for age and sex, and a height velocity below the 25th percentile—once other potentially treatable risk factors for growth failure have been adequately addressed and provided the child has growth potential” (13). “RhGH therapy should also be considered for children with stages 3–5 CKD or on dialysis aged above 6 months who present with a height between the 3rd and 10th percentile, but with persistent low height velocity (<25th percentile), once other potentially treatable risk factors for growth failure have been adequately addressed” (13). “Such early, preventive therapy is probably more cost-effective than starting at a more advanced age, when growth retardation has become more evident and higher absolute rhGH doses are required” (13). RhGH is substantially less effective in children requiring maintenance dialysis compared to children prior to dialysis. However, there is evidence that rhGH-induced catch-up growth can be markedly improved by intensified dialysis, e.g., by daily hemodialfiltration, which enhances dialytic clearance and thereby, probably, GH sensitivity (28, 46).

Children suffering from nephropathic cystinosis often show severe growth retardation despite somewhat mild reductions in GFR, which is thought to be related to the deleterious effects of Fanconi syndrome, resulting in hypophosphatemic rickets and malnutrition and/or an underlying obsteoblast/osteoclast defect (47). “Therefore, rhGH treatment is recommended in short children with nephropathic cystinosis in all stages of CKD” (47).

“Daily dosing is more effective than three doses per week and the optimal dose is 0.045–0.05 mg/kg body weight per day by subcutaneous injection in the evening” (13). “The primary treatment target should be to return the child's height to their individual genetic percentile channel” (13). “Treatment may be suspended once this target is reached, but growth should be further closely monitored” (13). “In rhGH treated patients with residual kidney function, rhGH should be continued after the initiation of dialysis, but stopped at the time of kidney transplantation” (13). “RhGH therapy should, however, subsequently also be considered for pediatric kidney transplant recipients for whom expected catch-up growth cannot be achieved by steroid minimization, or for patients in whom steroid withdrawal is not feasible due to high immunological risks, particularly in children with suboptimal graft function (GFR < 50 ml/min/1.73 m^2^) (vide infra)” (13). “Growth should be monitored for at least 1 year post-transplantation before rhGH therapy is considered, in order to allow for spontaneous catch-up growth without the use of rhGH therapy” (13).

### Transplantation

Kidney transplantation corrects many of the metabolic and endocrine disorders contributing to uremic growth failure. However, catch-up growth after kidney transplantation occurs far from regularly and is usually only noted in young children (48). Apart from transplant function, age and extent of growth retardation at the time of transplantation, in addition to corticosteroid dosage, are inversely correlated with growth rates after transplantation. Late steroid withdrawal in patients treated with a combination of a calcineurin inhibitor and mycophenolate mofetil resulted in improved growth rates in steroid-free patients compared to controls, which was not associated with increased rejection rates (49). However, substantial catch-up growth was noted in prepubertal patients only.

It is reasonable to assume that an early steroid-withdrawal, or even complete steroid-avoidance, will result in better growth outcome. In line with this, a retrospective analysis of post-transplant growth in 74 pediatric patients who had been weaned off steroids within 6 months of transplantation showed remarkable findings (4). Normal adult height (> −2 SD) was achieved in 94 and 80% of prepubertal and pubertal patients, respectively. Similarly, early steroid-withdrawal (<6 weeks) and complete steroid avoidance was shown to be safe and resulted in a height increment of ~1 SDS within 3–5 years after kidney transplantation (50–53). There is evidence that leg length is more reduced than trunk length in children requiring kidney replacement therapy, resulting in disproportionate stunting (39). Therefore, it is important to note that kidney transplantation results in preferential stimulation of leg growth in these patients and is thereby able to completely normalize body proportions by adulthood, if the transplant is performed before puberty (54). Thus, efforts to avoid a substantial height deficit before transplantation, through the use of rhGH treatment, early (preemptive) renal transplantation, and immunosuppressive strategies characterized by the early withdrawal, or even complete avoidance of glucocorticoid medication, should be undertaken to improve growth outcome and normalize body proportions in pediatric renal allograft recipients.

## Conclusions

Early diagnosis and adequate management of growth failure is of the utmost importance in children with CKD. The main measures are (i) preservation of kidney function by using renin-angiotensin aldosterone system inhibitors (RAAS), (ii) ensuring adequate energy intake, (iii) correction of acidosis and electrolyte imbalances, (iv) initiation of rhGH treatment in case of persistent growth failure, and (v) the provision of adequate dialysis in children on maintenance dialysis. Finally, as with any child with end-stage CKD, the ultimate goal is to perform (preemptive) kidney transplantation, in order to avoid the growth-suppressing effects of long-term dialysis and to provide enough GFR to allow for adequate growth.

## Author Contributions

The author confirms being the sole contributor of this work and has approved it for publication.

## Conflict of Interest

The author declares that the research was conducted in the absence of any commercial or financial relationships that could be construed as a potential conflict of interest.
